# Supplementary material for: How Does Lagenaria siceraria (Bottle Gourd) Metabolome Compare to Cucumis sativus (Cucumber) F. Cucurbitaceae? A Multiplex Approach of HR-UPLC/MS/MS and GC/MS Using Molecular Networking and Chemometrics
Source: Foods. 2023 Feb 10;12(4):771. doi: 10.3390/foods12040771 (PMC9956347; doi:10.3390/foods12040771)
Supplement: Supplementary file 1 [file foods-12-00771-s001.zip › foods-2190447-supplementary.pdf]

Table S1. Relative percentile of volatile constituents detected in *C. sativus* and *L. siceraria* fruits *via* SPME/GCMS

Peak	Rt (min.)	KI	Identification	*C. sativus*	*L. siceraria*
**Alcohols**					
1	7.743	1054	n-Octanol	0.24±0.003	0.41±0.05
2	7.94	1075	Dimethylbenzyl alcohol	0.25±0.001	0.22±0.04
3	8.07	1087	Linalool	17.7±0.300	18.76±0.33
4	8.259	1104	Phenylethyl Alcohol	0.15±0.001	0.68±0.15
5	8.465	1125	Terpineol <1->	0.89±0.890	1.41±0.12
6	8.947	1170	4-Terpineol	0.21±0.001	0.24±0.006
7	9.011	1175	*p*-Cymen-8-ol	0.21±0.010	0.26±0.03
8	9.076	1182	alpha-Terpineol	0.83±0.002	0.67±0.03
9	9.551	1229	cis-*ρ*-Mentha-1(7),8-dien-2-ol	0.34±0.029	0.2±0.014
10	12.832	1561	Tridecanol <n->	0.18±0.000	0.2±0.034
11	15.145	1810	Phytol	0.19±0.008	0.23±0.13
			**Total Alcohols**	**21.27**	**23.34**
**Aldehydes**					
12	5.065	803	2-Hexenal	1.03±0.213	1.21±0.48
13	6.403	929	2-Heptenal	0.17±0.023	0.42±0.11
14	6.421	937.2	Benzaldehyde	2.59±0.080	1.93±0.55
15	6.858	983	Octanal	11.1±0.029	9.18±1.31
16	7.067	994	Heptadienal <(2E,4E)->	0.27±0.003	0.41±0.067
17	7.478	1031	Benzeneacetaldehyde	3.60±0.043	1.39±0.25
18	8.11	1091	Nonanal	1.84±0.063	1.71±0.31
19	8.631	1140	2,6-Nonadienal	1.60±0.014	0.22±0.019
20	8.718	1147	2-Nonenal	1.38±0.046	1.38±0.02
21	9.159	1189	Decanal	1.03±0.109	0.96±0.1
22	9.694	1247	m-Methoxybenzaldehyde	0.03±0.000	0.025±0.003
23	9.884	1263	Cinnamaldehyde	0.39±0.003	0.45±0.087
24	10.261	1304	2,4-Decadienal	0.14±0.028	0.17±0.11
25	12.407	1520	Dodecadienal <(2E,4E)->	0.12±0.002	0.13±0.029
26	14.181	1704	Pentadecanal-	0.04±1.676	0.15±0.043
27	15.193	1815	4,8,12-Tetradecatrienal, 5,9,13-trimethyl-	0.20±0.045	0.22±0.081
			**Total Aldehydes**	**25.63**	**20.04**
**Aliphatic hydrocarbons**					
28	8.906	1158	Dodecene	0.03±0.002	0.03±0.003
29	9.238	1196	Dimethyl undecane	0.04±0.001	0.01±0.001
30	9.894	1265	4,6-Dimethyldodecane	0.41±0.019	0.39±0.01
31	10.317	1310	Trimethyl dodecane	0.26±0.017	0.24±0.01
32	10.97	1379	Tetradecane	0.56±0.005	0.48±0.05
33	12.274	1508	n-Pentadecane	0.11±0.002	0.11±0.01
34	12.915	1569	n-Hexadecane	0.24±0.018	0.278±0.04
			**Total Aliphatic hydrocarbons**	**1.69**	**1.55**
**Aromatic hydrocarbons**					
35	7.992	1080	Dimethylstyrene	1.04±0.046	0.95±0.006
36	9.065	1180	Naphthalene	1.07±0.097	1±0.12
37	10.332	1311	Methyl naphthalene	0.14±0.007	0.14±0.008
			**Total Aromatic hydrocarbons**	**2.27**	**2.10**

Continue Supplementary Table S1.

Peak	Rt (min.)	KI	Identification	*C. sativus*	*L. siceraria*
**Esters**					
38	8.416	1109	Methyl octanoate	1.73±0.048	1.41±0.1
39	8.999	1174	Butyl caproate	0.49±0.025	0.42±0.05
40	9.197	1192	n-Octyl acetate	0.04±0.004	0.033±0.003
41	9.332	1205	Methyl heptanoate	0.21±0.009	0.11±0.02
42	9.396	1212	Propylpentyl acrylate	0.17±0.003	0.18±0.04
43	9.651	1240	Linalool acetate	3.67±0.303	3.09±0.47
44	9.688	1243	Isoamyl hexanoate	0.12±0.003	0.12±0.02
45	9.95	1273	Allyl octanoate	0.85±0.087	0.75±0.04
46	10.148	1292	n-Nonanyl acetate	0.22±0.001	0.22±0.004
47	10.559	1336	Methyl o-aminobenzoate	0.13±0.006	0.16±0.005
48	10.593	1339	alpha-Terpineol acetate	1.51±0.054	1.58±0.1
49	10.663	1347	Nerol acetate	0.40±0.001	0.4±0.03
50	10.866	1368	Butyl caprylate	0.03±0.00	0.047±0.01
51	11.172	1400	Methyl N-methyl anthranilate	0.17±0.002	0.17±0.009
52	13.632	1645	Methyl jasmonate	0.05±0.008	0.047±0.009
53	14.244	1711	Methyl tetradecanoate	0.10±0.000	0.12±0.017
54	14.68	1759	Benzyl benzoate	0.04±0.015	0.1±0.03
55	15.018	1797	Isopropyl Myristate	0.00±0.000	0.14±0.04
56	15.03	1798	Pentadecanoic acid, methyl ester	0.27±0.083	0.41±0.09
57	15.35	1832	Phytol acetate <(E)->	0.07±0.006	0.08±0.03
58	15.885	1887	Methyl hexadecanoate	0.45±0.026	1.6±0.37
59	17.809	2087	Methyl linoleate	0.14±0.027	0.35±0.06
			**Total Esters**	**10.97**	**11.64**
**Fatty acids**					
60	16.187	1918	n-Hexadecanoic acid	0.05±0.013	0.13±0.05
61	17.886	2095	Oleic acid	0.17±0.036	0.23±0.02
62	15.226	1818	Unknown	0.02±0.003	0.026±0.01
			**Total Fatty acids**	**0.26**	**0.39**
**Ketones**					
64	6.745	964.3	6-Methyl-5-Hepten-2-one	0.06±0.062	0.18±0.01
65	7.766	1056	Acetophenone	0.00±0.000	0.02±0.01
66	7.786	1057	3,5-Octadien-2-one	0.08±0.003	0.05±0.009
67	8.008	1081	Fenchone	0.70±0.004	3.15±4.17
68	8.722	1147.2	*p*-Menthone	0.19±0.010	0.2±0.1
69	8.827	1157	Iso p-Menthone	0.77±0.021	0.77±0.01
70	9.354	1208	4-methylene-Isophorone	0.16±0.006	0.14±0.01
71	9.617	1236	Carvone	0.33±0.010	0.39±0.003
72	9.719	1248	*p*-menth-1-en-3-one (Piperitone)	0.25±0.005	0.23±0.03
73	9.75	1250	Benzyl isopropyl ketone	0.07±0.004	0.08±0.03
74	11.232	1405	dihydro-α-Ionone	0.05±0.002	0.06±0.003
75	11.47	1429	Geranyl acetone	0.26±0.019	0.34±0.07
76	11.805	1462	Ionone <isomethyl-α-(E)->	6.59±0.053	6.87±1.03
77	12.011	1481	Ionone <methyl-γ->	0.25±0.003	0.26±0.04
			**Total Ketones**	**9.84**	**12.81**

Continue Supplementary Table S1.

Peak	Rt (min.)	KI	Identification	*C. sativus*	*L. siceraria*
**Monoterpene hydrocarbons**					
78	7.251	1011	*p*-Cymene	0.65±0.010	0.81±0.02
79	7.303	1015	Limonene	1.51±0.030	1.5±0.03
80	7.639	1048	gamma-terpinene	0.35±0.006	0.33±0.005
			**Total Monoterpene hydrocarbons**	**2.53**	**2.66**
**Oxides/ethers**					
81	7.344	1019	Cineole <1,8	1.46±0.049	1.92±0.13
82	9.035	1178	Dill ether	0.46±0.024	0.54±0.02
83	10.01	1277	Anethol	11.2±1.050	9.43±1.64
84	10.06	1283	Safrole	10.5±0.643	11.4±0.95
85	10.527	1332	Isosafrole <(Z)->	0.02±0.002	0.037±0.02
86	10.616	1341	Eugenol	0.06±0.005	0.049±0.0009
87	10.878	1369	Isosafrole <(E)->	0.07±0.007	0.11±0.055
			**Total Oxides/ethers**	**23.93**	**23.52**
**S-containing compounds**					
88	5.707	874	Methional	0.02±0.006	0.007±0.004
			**Total S-containing compounds**	**0.02**	**0.01**
**Sesquiterpene hydrocarbons**					
89	10.906	1371	Copaene	0.18±0.000	0.18±0.016
90	11.335	1416	Caryophyllene	0.09±0.002	0.11±0.017
91	11.509	1432	β-Farnesene (E)	0.24±0.015	0.22±0.025
92	12.049	1485	α-Amorphene	0.28±0.014	0.3±0.06
93	12.227	1504	Farnesene <(E,E)-α->	0.25±0.016	0.22±0.06
			**Total Sesquiterpene hydrocarbons**	**1.07**	**1.05**

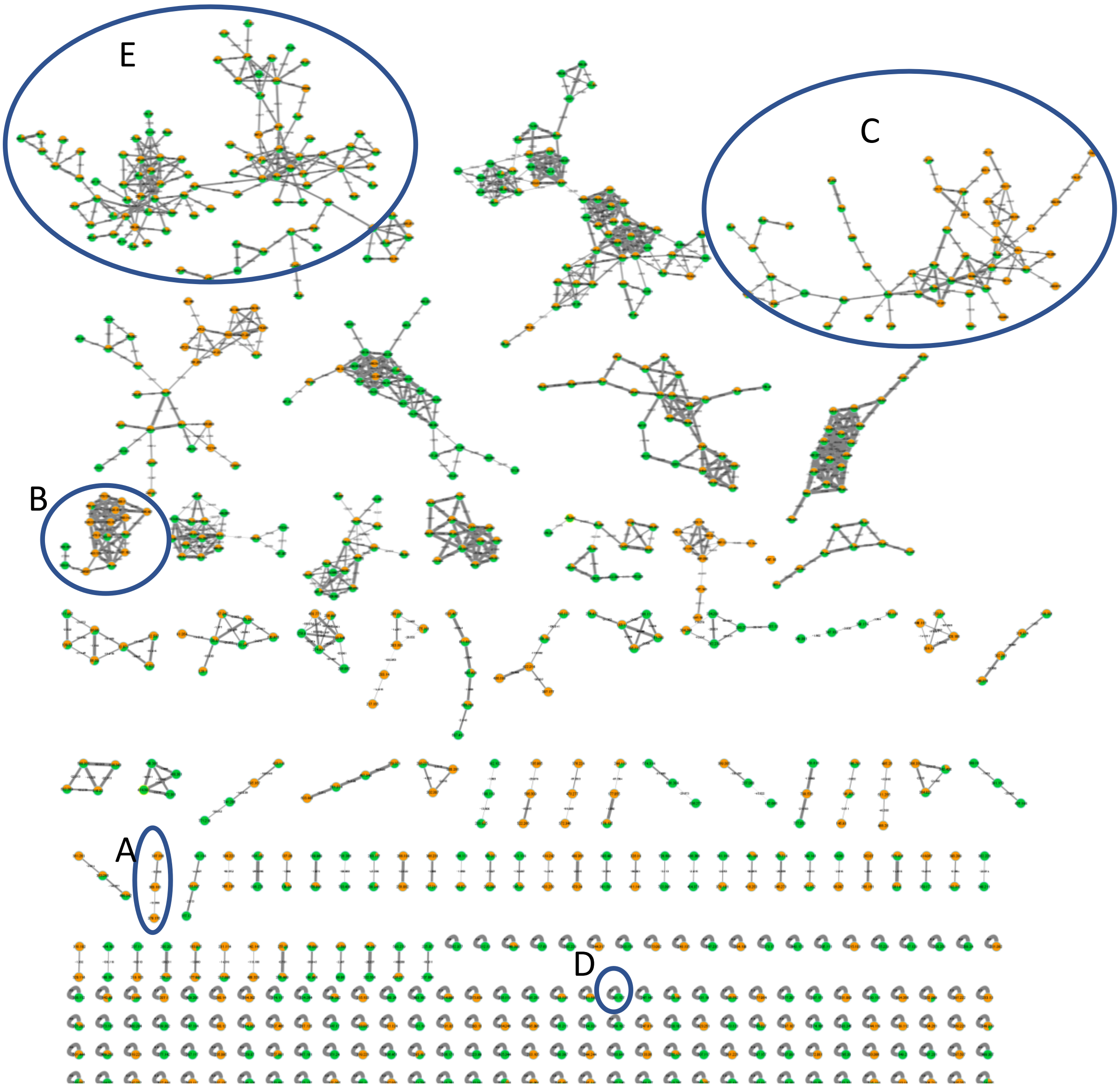

Figure S1. Full molecular networking created using MS/MS data in positive ionization mode for *L. siceraria* (bottle gourd) and *C. sativa* (cucumber) crude fruit extracts showing 1002 nodes and 1451 edges. All nodes are labeled with parent mass and edges are labelled with neutral loss values. The network is displayed as pie chart with orange and green colors representing distribution of the precursor ion intensity in the pumpkin and cucumber extracts respectively.

Clusters annotation: **A**: Lignans, **B:** Flavonoids, **C**: Amino acids derivatives, **D**: Alkaloid (singleton), **E**: Fatty acids/amides

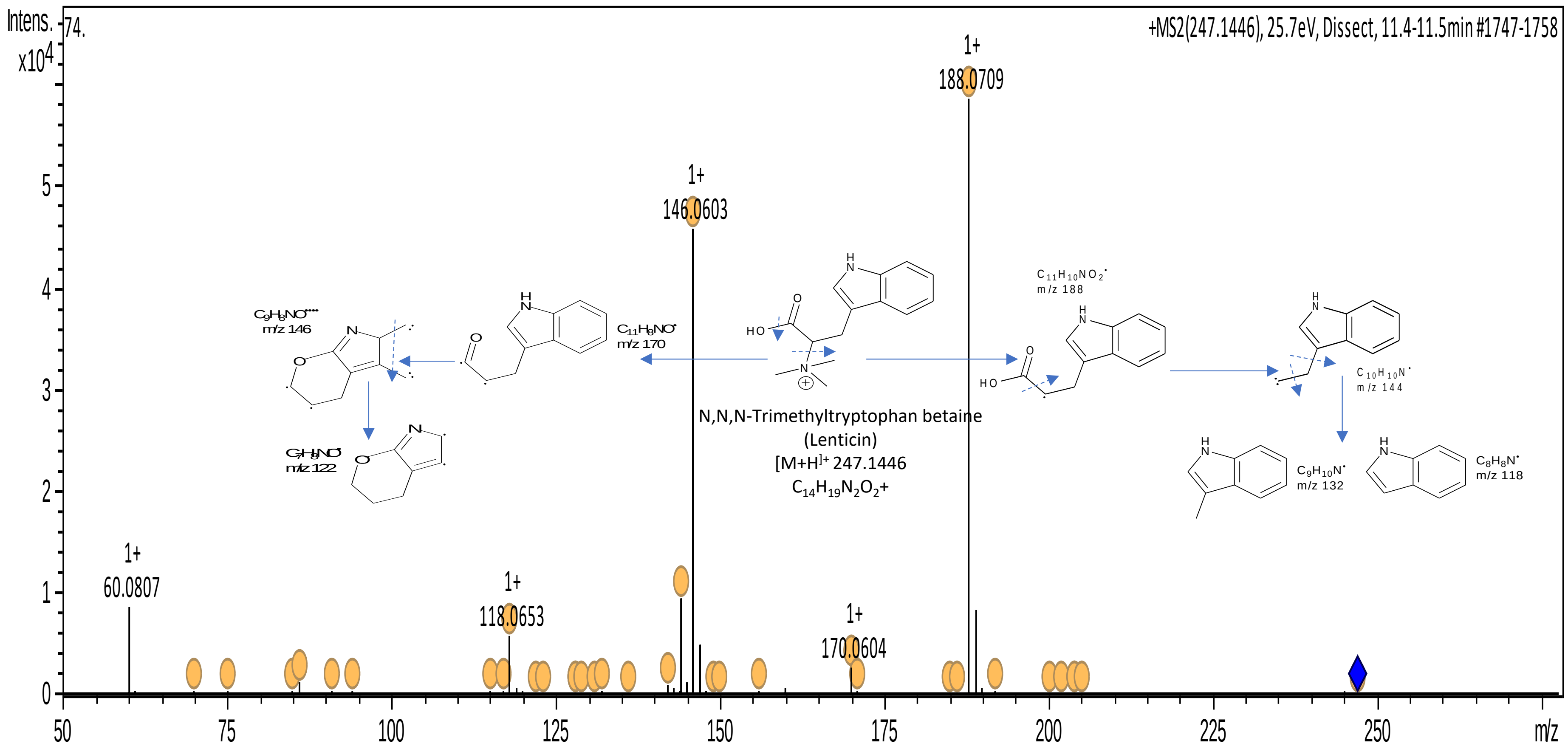

Figure S2. Tandem MS/MS spectrum of N,N,N-Trimethyltryptophan betaine (or Lenticin) alkaloid (peak 16) detected in *C. sativa* fruit crude extract *via* HR-UPLC/MS/MS analysis in positive ionization mode.

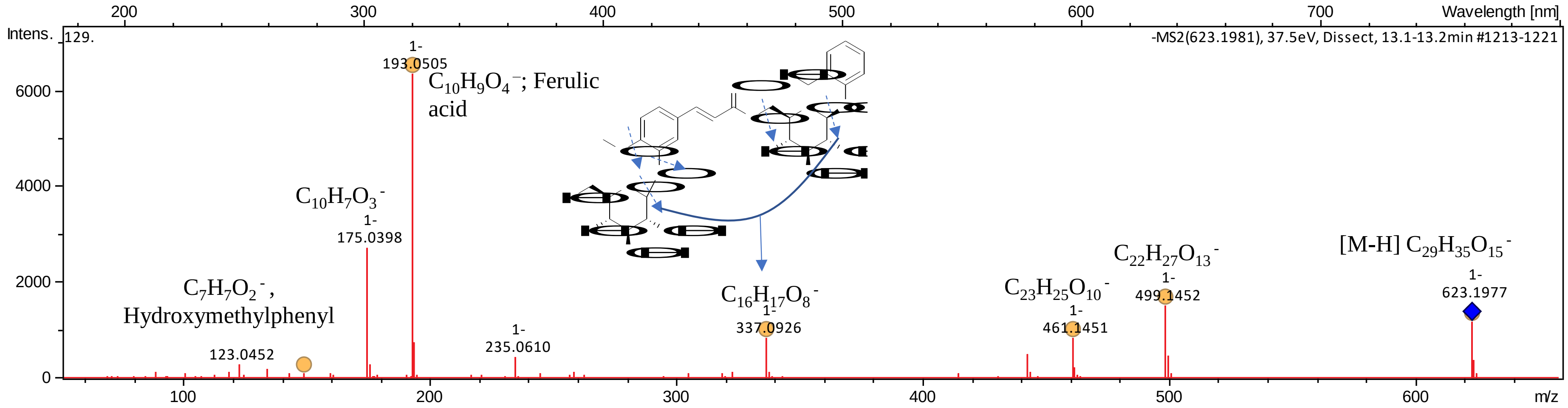

Figure S3. Tandem MS/MS spectrum of hydroxymethylphenyl-*O*-hexosylferuloyl-*O*-hexoside (peak 29) detected in *L. siceraria* fruit crude extract *via* HR-UPLC/MS/MS analysis in negative ionization mode.

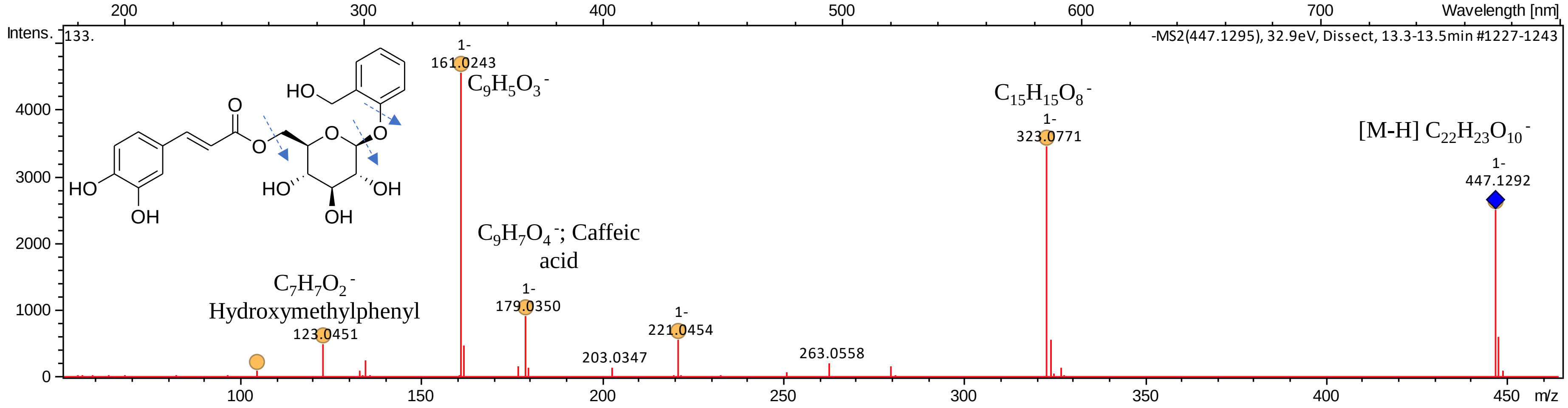

Figure S4. Tandem MS/MS spectrum of hydroxymethylphenyl -*O*-caffeoyl-*O*-hexoside (peak 31) detected in *L. siceraria* fruit crude extract *via* HR-UPLC/MS/MS analysis in negative ionization mode.

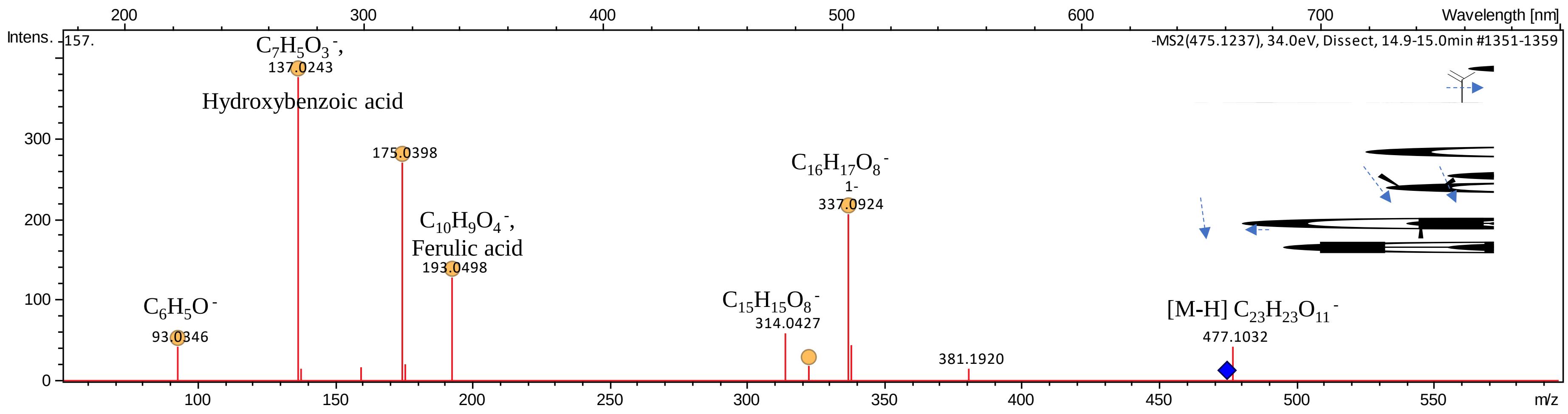

Figure S5. Tandem MS/MS spectrum of hydroxybenzoic acid -*O*-feruloyl-*O*-hexoside (peak 36) detected in *L. siceraria* fruit crude extract *via* HR-UPLC/MS/MS analysis in negative ionization mode.

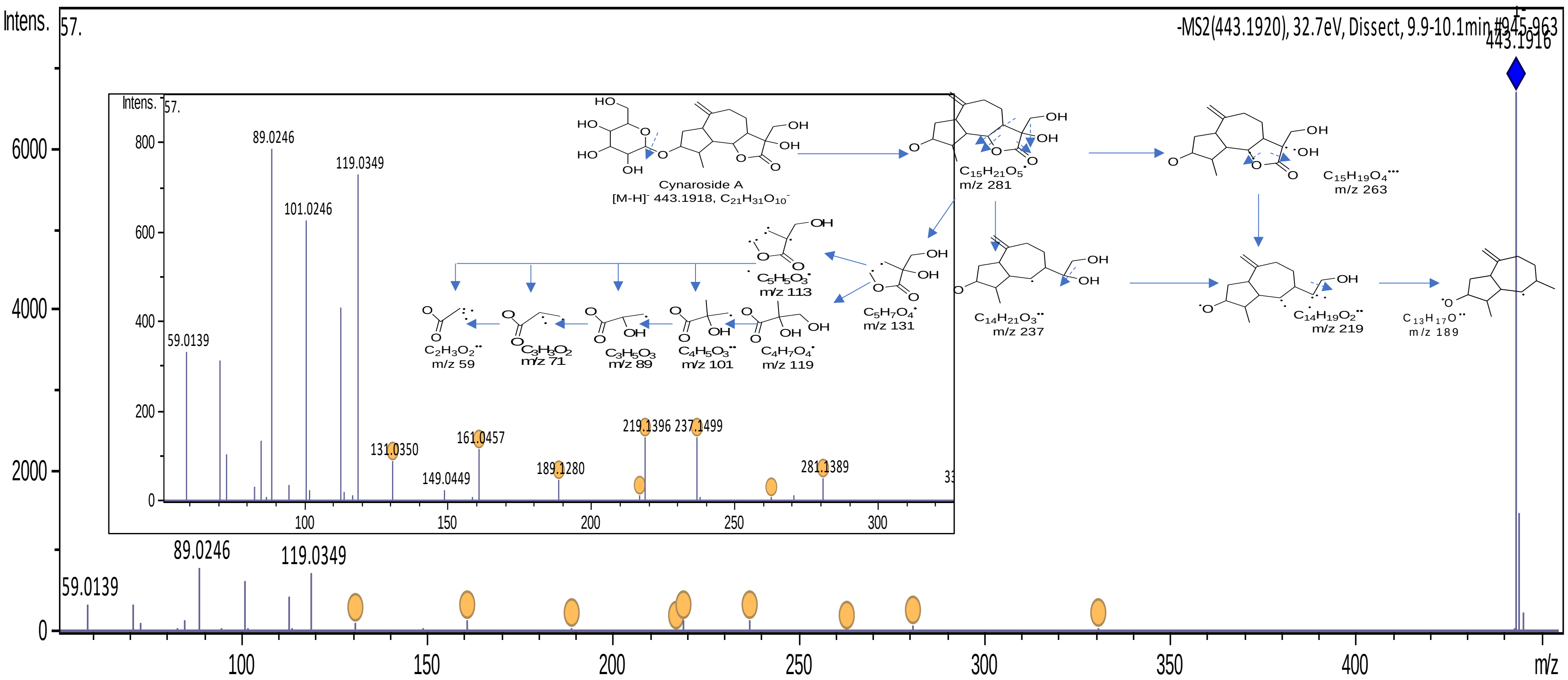

Figure S6. Tandem MS/MS spectrum of cynaroside A sesquiterpene (peak 39) detected in both *L. siceraria* & *C. sativa* fruit crude extracts *via* HR-UPLC/MS/MS analysis in negative ionization mode.

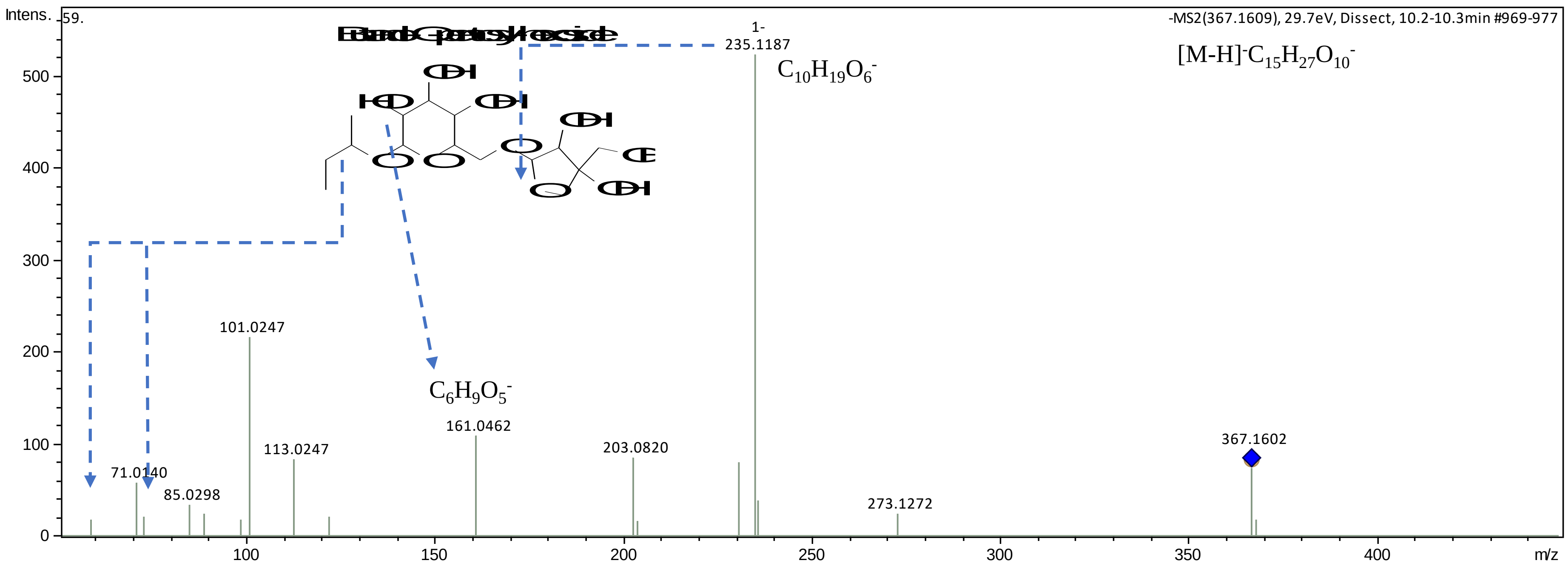

Figure S7. Tandem MS/MS spectrum of butanol-O-pentosyl-hexoside (peak 44) detected in *C. sativa* fruit crude extract *via* HR-UPLC/MS/MS analysis in negative ionization mode.

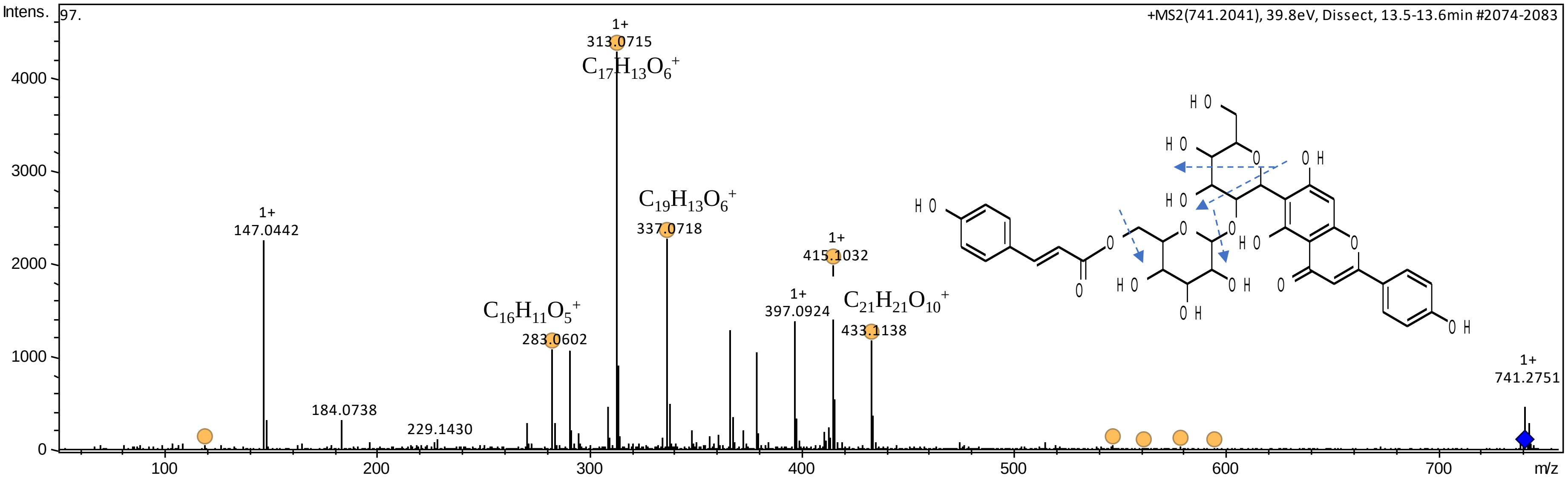

Figure S8. Tandem MS/MS spectrum of iso/vitexin-*O*-couamroylhexoside (peak 54) detected in *C. sativa* fruit crude extract *via* HR-UPLC/MS/MS analysis in positive ionization mode.

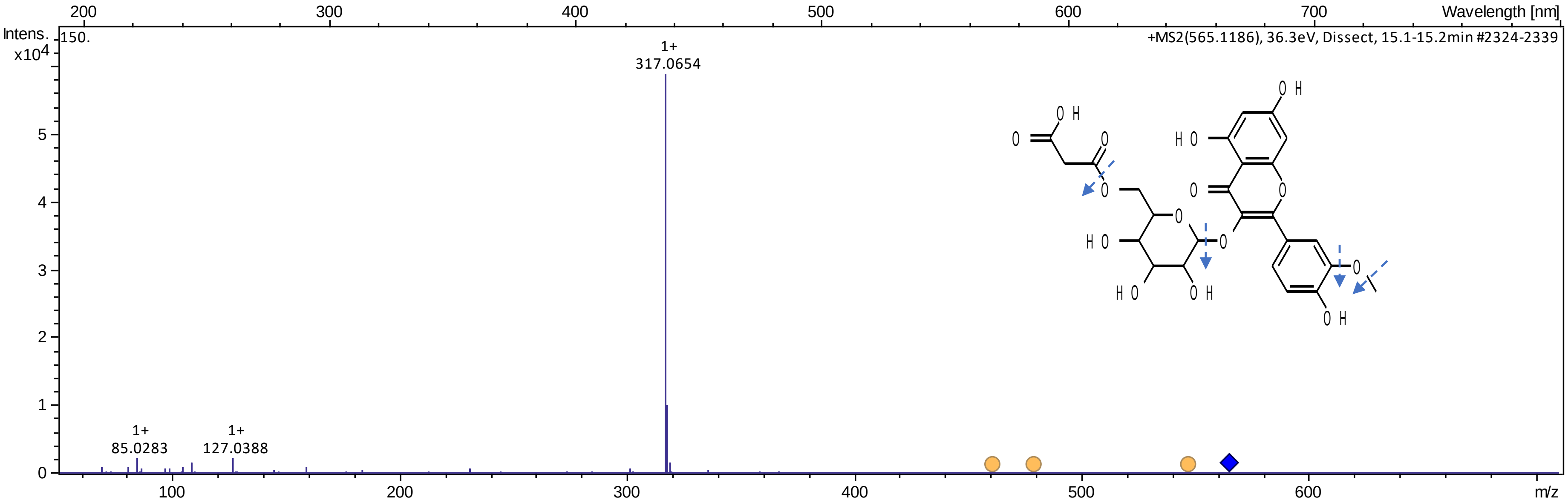

Figure S9. Tandem MS/MS spectrum of isorhamnetin-O-malonylhexoside (peak 62) detected in *L. siceraria* & *C. sativa* fruit crude extract *via* HR-UPLC/MS/MS analysis in positive ionization mode.

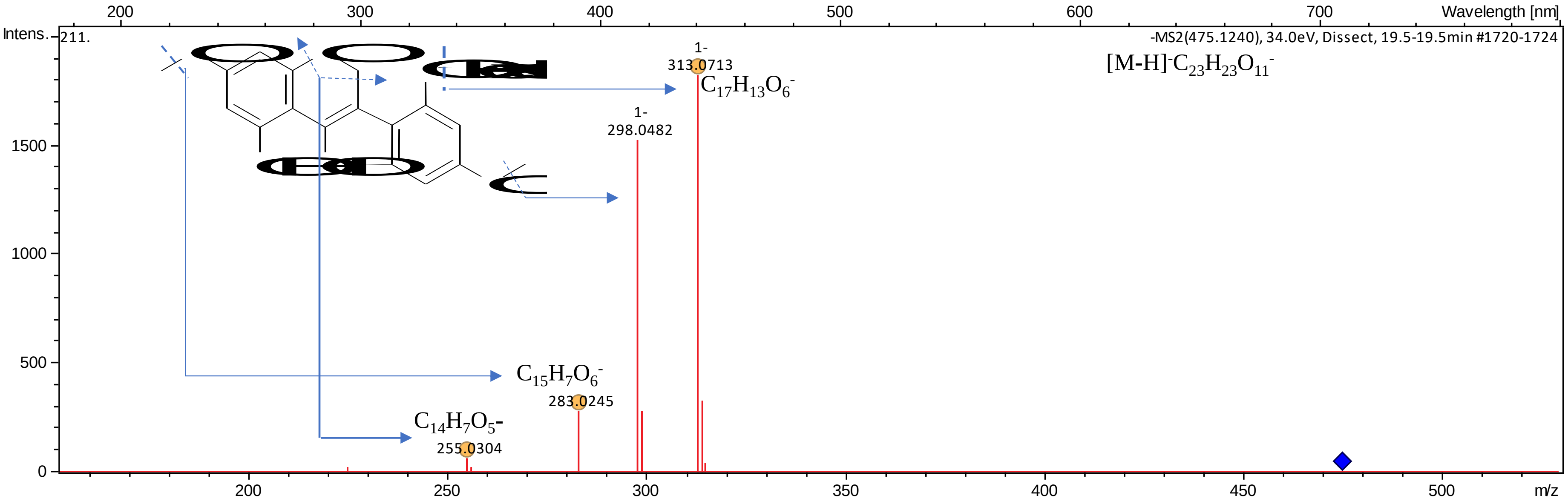

Figure S10. Tandem MS/MS spectrum of hedysarimpterocarpene A-*O*-hexoside or HPA-*O*-hexoside (peak 72) detected in *C. sativa* fruit crude extract *via* HR-UPLC/MS/MS analysis in negative ionization mode.

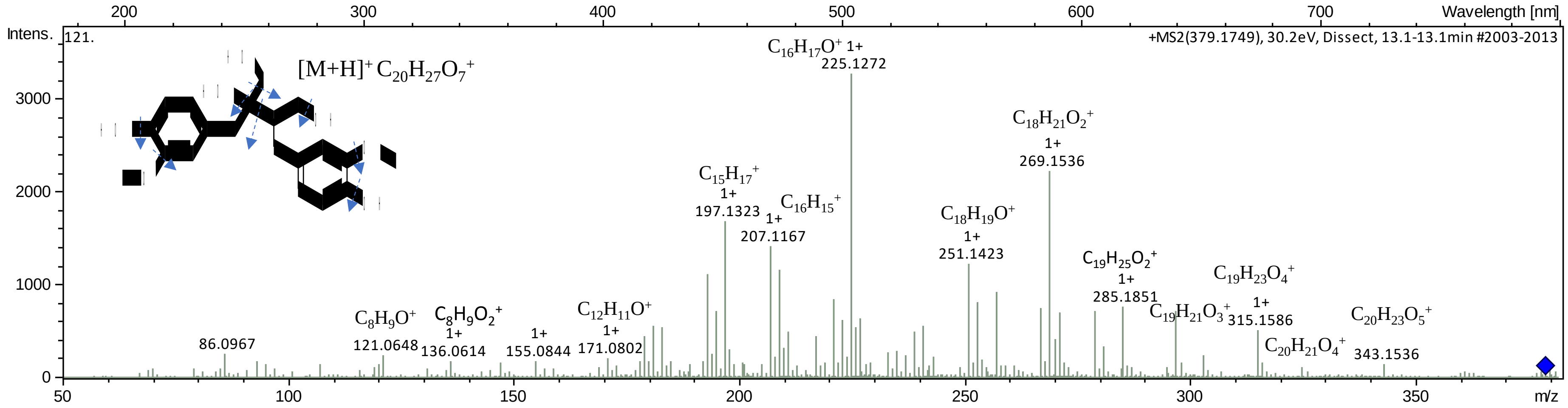

Figure S11. Tandem MS/MS spectrum of pentahydroxy-dimethoxylignan (peak 75) detected in *C. sativa* fruit crude extract *via* HR-UPLC/MS/MS analysis in positive ionization mode.

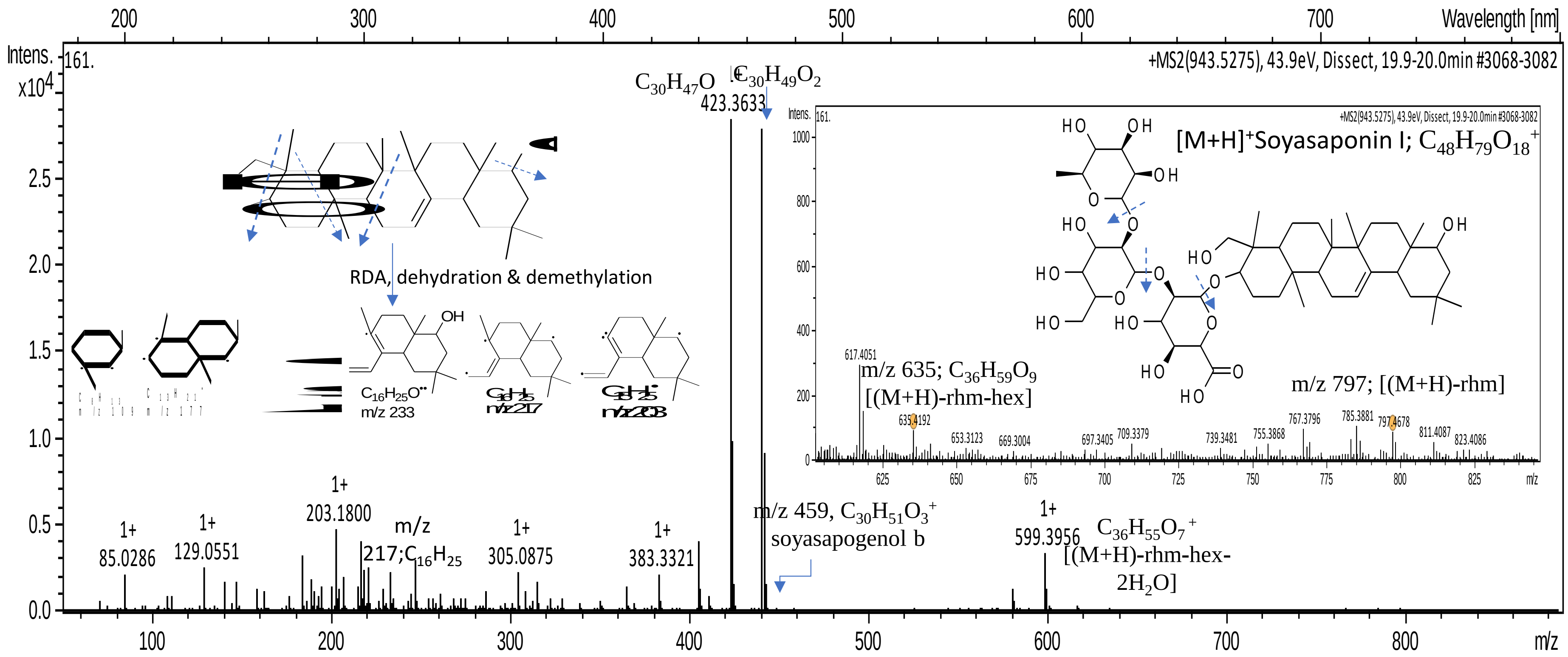

Figure S12. Tandem MS/MS spectrum of soyasaponin I (peak 82) detected in *C. sativa* fruit crude extract *via* HR-UPLC/MS/MS analysis in positive ionization mode.

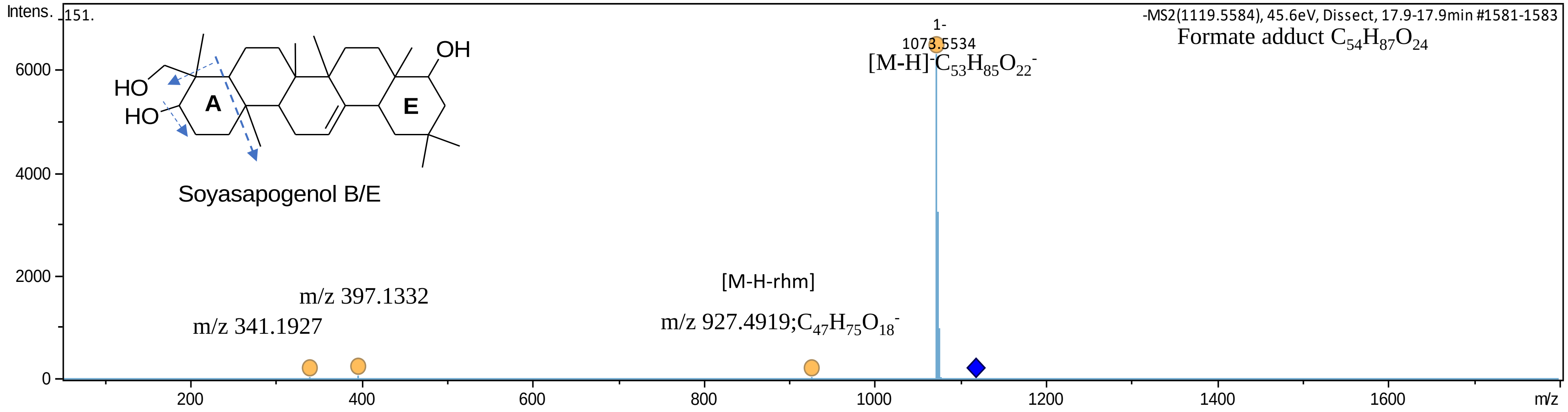

Figure S13. Tandem MS/MS spectrum of melilotussaponin O1(peak 81) detected as formate adducts in *C. sativa* fruit crude extract *via* HR-UPLC/MS/MS analysis in negative ionization mode and identified based on GNPS networking with soyasaponin I in cluster E.

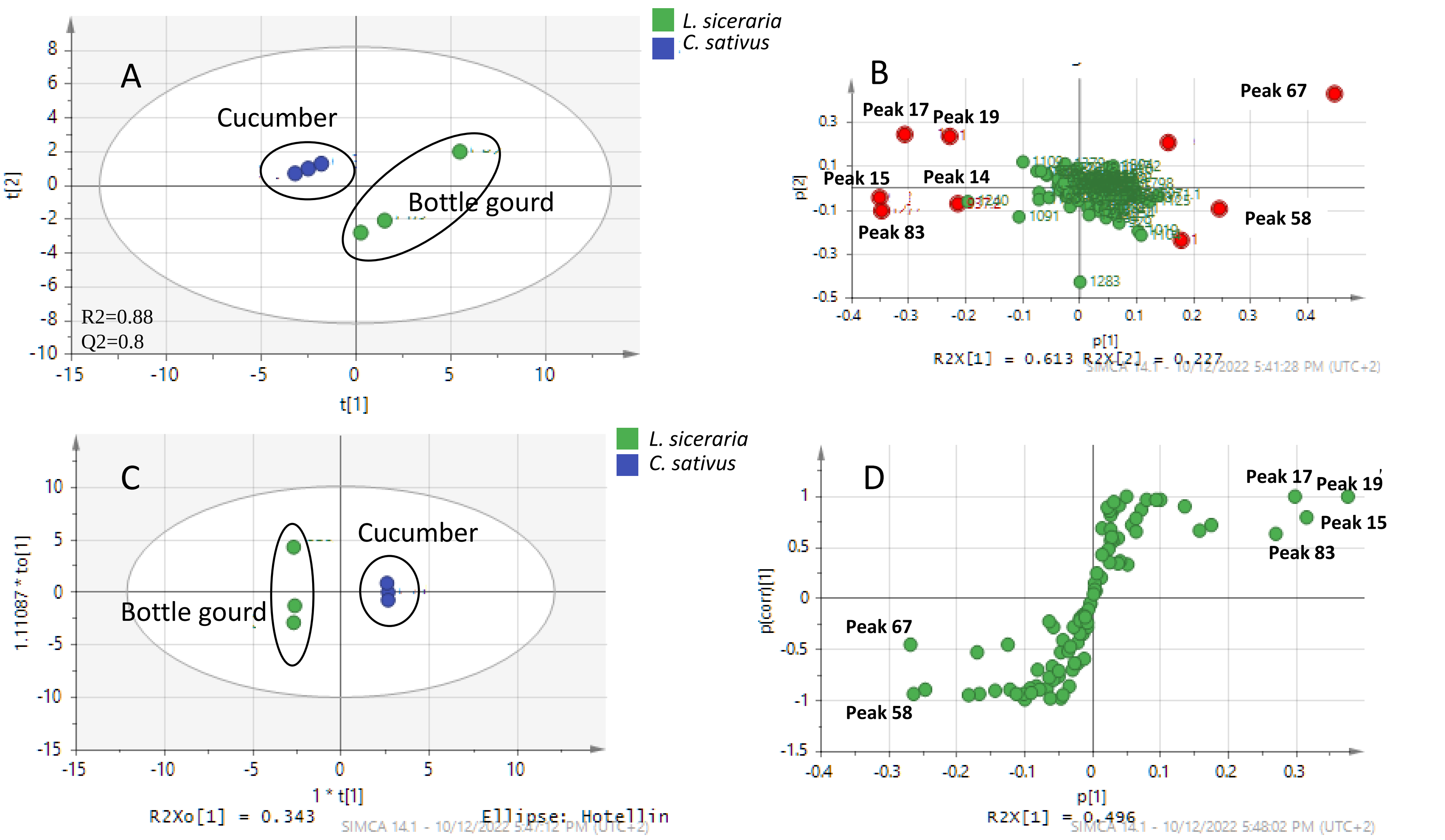

Figure S14. Principal component analysis (PCA) and orthogonal projection to latent structures-discriminant analysis (OPLS) supervised data analysis of modelling *C. sativus* and *L. siceraria* fruit specimens analyzed via SPME GC-MS for their volatile metabolites ($n = 3$). PCA score (A) and loading plot (B) with PC1=61% and PC2=22%; OPLS-DA score plot (C) and loading S-plot (D). Variables labelled with peak numbers (as in Table S1) correspond to discriminating metabolites for each sample identified by their Mol.wt/Rt. Benzaldhyde (peak 14), octanal (peak 15), benzenacetaldehyde (peak 17), nonadienal (peak 19), anethol ether (peak 83), fenchone (peak 67) and methyl hexadecanoate (peak 58) are the discriminating biomarkers.

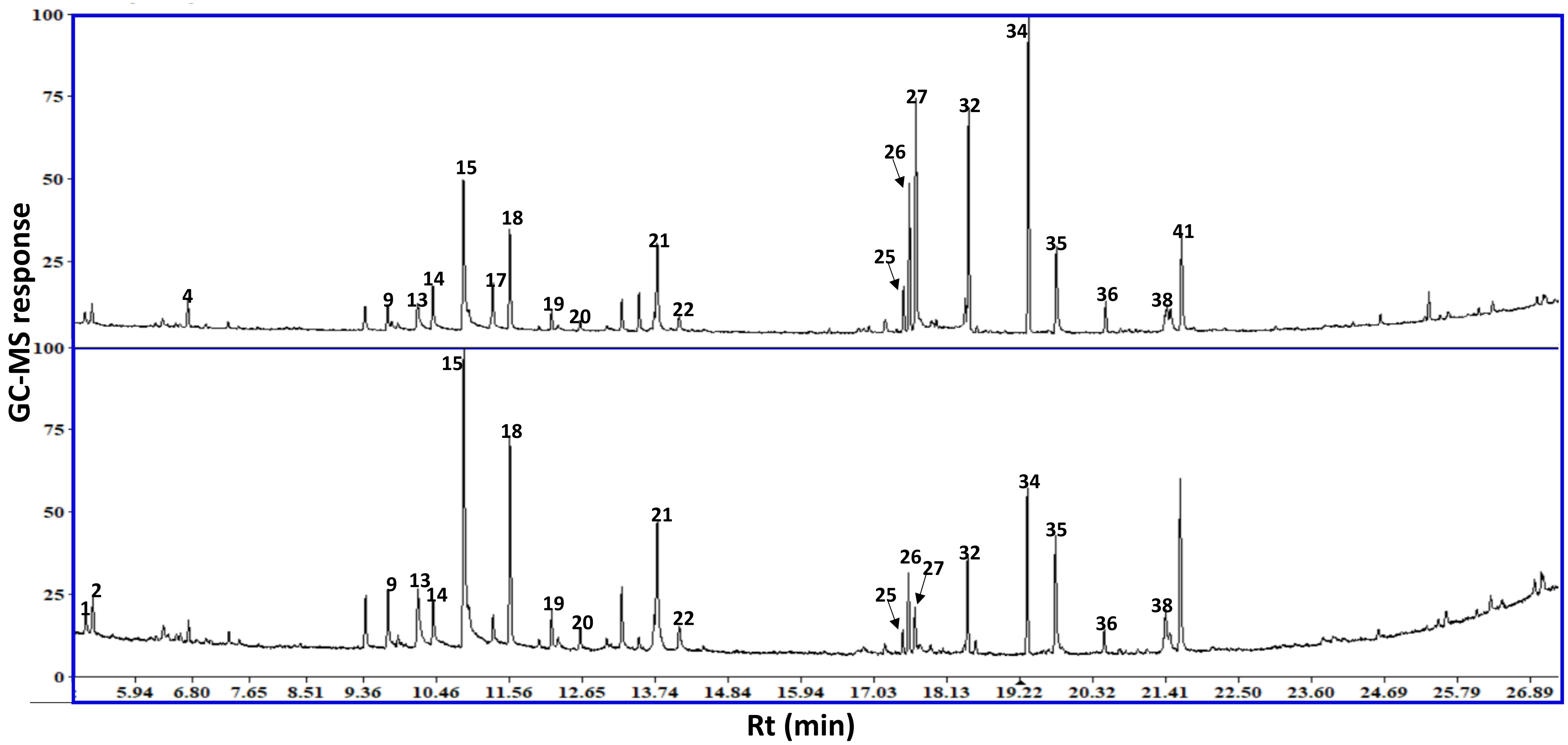

Figure S15. Representative GC-MS chromatograms of *C. sativus* and *L. siceraria* fruit specimens' silylated primary metabolites. Assigned peak numbers follow those shown in **Table 2**.

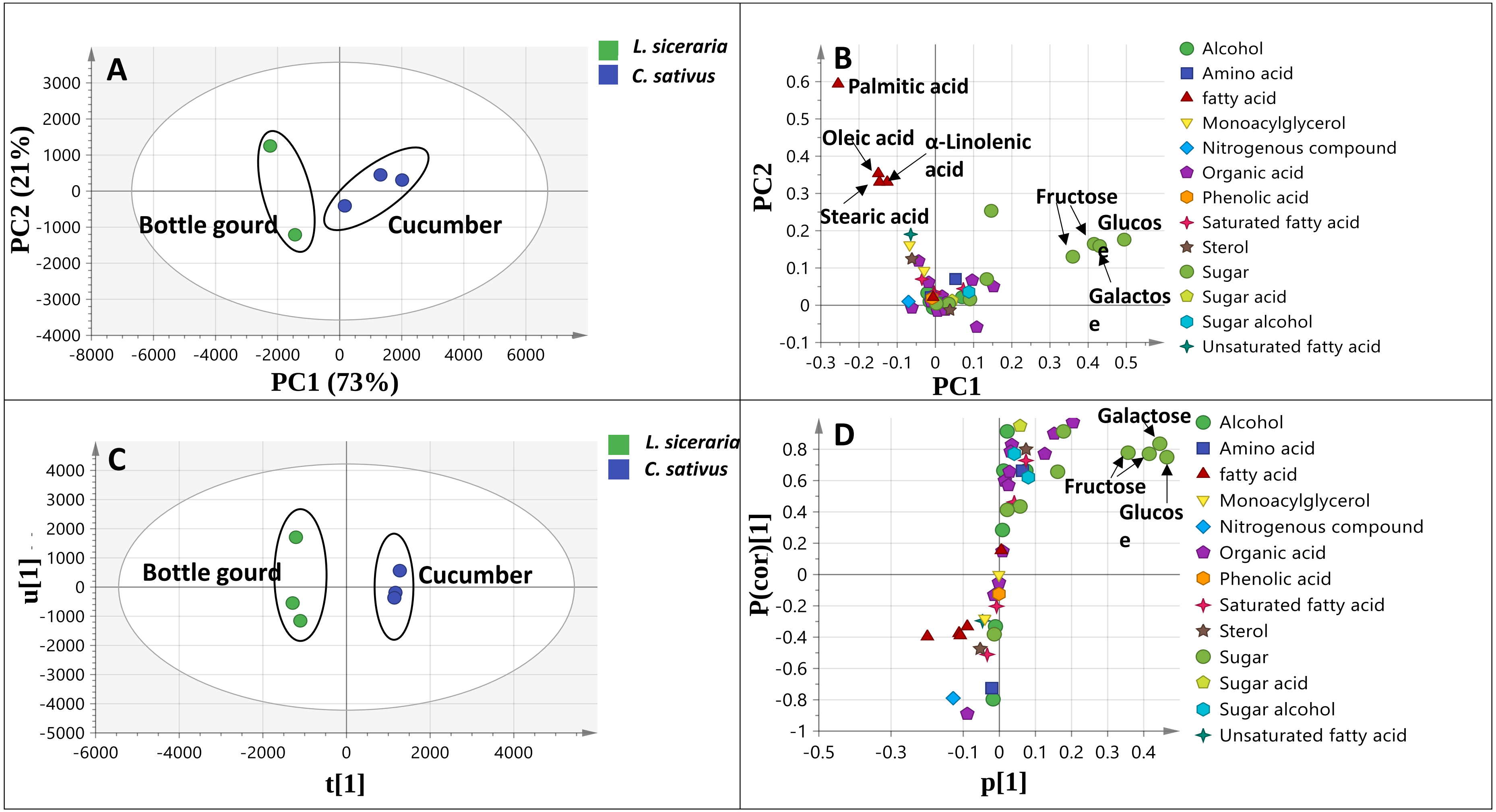

Figure S16. Principal component analysis (PCA) and orthogonal projection to latent structures-discriminant analysis (OPLS) supervised data analysis of modelling *C. sativus* and *L. siceraria* fruit specimens analyzed *via* GC-MS for their silylated primary metabolites (n = 3). PCA score (A) and loading plot (B) with PC1=73% and PC2=21%; OPLS-DA score plot (C) and loading S-plot (D). Variables labelled with their names follow those listed in **Table 2**.
